# COVID-19 outbreak and dental health care provision in Nigeria: a national survey

**DOI:** 10.1186/s12903-021-01860-1

**Published:** 2021-10-04

**Authors:** Timothy Olukunle Aladelusi, Foluso Ayobami Atiba, Shakeerah Olaide Gbadebo, Yewande Isabella Adeyemo, Adeola Adenike Olusanya, Oladimeji Adeniyi Akadiri

**Affiliations:** 1 Department of Oral and Maxillofacial Surgery, College of Medicine, University of Ibadan, Ibadan, Oyo State Nigeria; 2 Department of Anatomy, College of Medicine, University of Ibadan, Ibadan, Oyo State Nigeria; 3 Department of Restorative Dentistry, College of Medicine, University of Ibadan, Ibadan, Oyo State Nigeria; 4 Department of Child Dental Health, Faculty of Dentistry, College of Health Sciences, Bayero University, Kano, Kano State Nigeria; 5 Department of Oral and Maxillofacial Surgery, College of Health Sciences, University of Port Harcourt, Port Harcourt, Rivers State Nigeria

**Keywords:** COVID-19, Dental care delivery, Infection control, Personal protective equipment, SARS-CoV-2

## Abstract

**Background:**

The impact of the COVID-19 pandemic on the world is unprecedented, posing greater threats to vulnerable healthcare systems, especially in developing countries. This study aimed to assess the knowledge of dental healthcare providers in Nigeria about the disease and evaluate their responses to the preventive measures necessitated by COVID-19.

**Methods:**

This was an online self-administered questionnaire-based study conducted among dentists practicing in Nigeria. A message containing the link to the survey was sent widely via social medial platforms and electronic mails to dentists practicing in Nigeria. The data collection was done between the 2^nd^ of June and 3rd of July 2020.

**Results:**

A total of 314 responses was recorded. Fever was the most specified generalized symptom (97.5%), while the use of masks (100%), hand hygiene (99.7%), social distancing (97.7%) and surface cleaning (99.4%) were the most commonly employed general preventive methods. The main identified risk of transmission in the clinic was aerosol generating procedures (98.7%).

**Conclusion:**

The general knowledge of dental personnel in our study population appears to be adequate on the common clinical features of COVID-19 but less adequate regarding the less common features. The COVID-19 pandemic has also modified some aspects of dental service delivery but more needs to be done in this regard. Preventive measures against the transmission of COVID-19 in dental practice settings include proper utilization of teledentistry, clinical triage, preprocedural 1% hydrogen peroxide oral rinses, and the use of appropriate Personal Protective Equipment (PPE) which should always be encouraged.

## Background

The world had experienced several viral epidemics (such as the highly pathogenic influenza virus (H5N1), swine flu (H1N1), avian influenza, Severe Acute Respiratory Syndrome (SARS), Middle East Respiratory Syndrome (MERS), Ebola, and Zika) which were successfully combated by appropriate preventive public health measures and targeted research[1, 2]. However, the occurrence of new human pathogens and re-emergence of some previously controlled infectious diseases are of actual concern[1, 3].

A novel human coronavirus which was originally reported in Wuhan, China in December, 2019, now designated as severe acute respiratory syndrome coronavirus 2 (SARS-CoV-2), is responsible for the outbreak currently plaguing the world[4, 5]. This has been an unprecedented situation with severe economic implications across the globe[6–8]. On the 30th of January 2020, the World Health Organization (WHO) declared the outbreak of COVID-19 a public health emergency of international concern because of its widespread affectation and high risk to countries with weak health systems. Thereafter, on the 11th of March 2020, the disease was classified to be a pandemic. According to the WHO situation report of June 28, 2020 update on COVID-19, over 9,843,073 cases and 495,760 deaths have been reported worldwide[9].

Despite the vulnerable healthcare system in Nigeria, the government has been able to curtail the disease by imposition of partial lockdown and universal use of facemasks in public spaces; however, the number of reported cases in Nigeria was 25,694, with approximately 590 deaths as of the end of June, 2020 and these numbers have continued to rise with evidence of spread of COVID-19 in the community[10]. Given the continuous transmission of SARS-CoV-2, healthcare providers are at a higher risk of contracting the infection and becoming carriers of the disease. According to the Occupational Safety and Health Administration (OSHA), dental health care personnel (DHCP) are placed in “very high exposure risk” category[11–13] because they work very close to and around patients’ aero-digestive orifices for extended periods. Dental procedures involve face to face interactions and regular exposure of the oral health care teams to blood, saliva, and other body fluids[13]. Additionally, the frequent use of sharp and rotary instruments such as scalers and handpieces, which are aerosol generating, put the DHCP at a high risk of contracting SARS-CoV-2 or/and passing the infection to their patients[14]. Therefore, adequate understanding of the nature of the virus, clinical features, modes of transmission, and appropriate preventive measures among DHCP are all of paramount importance to guide protocols for dental practices and enhance prompt identification of cases for prevention of spread to the patients and the DHCP. Documentation of the responses of the healthcare system will also help us model similar response for future threats. This study aimed to assess the knowledge of dentists in Nigeria about COVID-19 and evaluate their responses to the preventive measures necessitated by the disease.. The impact of government-imposed lockdown among dentists was also documented. This information will help us understand the specific professional response to the pandemic in our environment and assist in planning response to future pandemics.

## Methods

This was an open survey, using an online self-administered questionnaire among dentists practicing in Nigeria. The sample size was calculated using the WINPEPI software to estimate the prevalence of impact of COVID-19 outbreak on dental healthcare service provision. Using WINPEPI computer programme for epidemiologists[15], an assumed prevalence of 15% with a two-sided alpha of 5%, a difference of 4% and a non-response rate of 10% yielded a minimum sample size of 309 respondents. Ethical clearance was obtained from the Oyo State Ministry of Health Ethics Committee (AD13/479/2003B).

A convenience sampling technique was used and the message containing the link to the survey was widely circulated via the National Dental Association and specialty social medial platforms and individualised electronic mails to dentists practicing in Nigeria. The data collection was done between the 2nd of June and 3rd of July 2020 at the height of the first wave of the pandemic in our country and respondents were advised to send in only one response. This was enforced by setting the survey to accept only one response per device. Consent to participate in the study was contained in the first question in the questionnaire and the participants were informed of the purpose of the study and the estimated length of time required to complete the questionnaire.

The questionnaire was developed and pretested for content and face validity by the researchers based on previous surveys on the same question. It was anonymised, personal information such as name or identification number were not recorded, mandatory questions were clearly specified to ensure internal consistency and non-response to such questions prevented further participation in the study. Cronbach’s Alpha value of the 16 items that assessed changes in practice necessitated by COVID-19 was 0.712 indicating acceptable reliability of the questionnaire. After three rounds of corrections, the questionnaire was piloted among 10 randomly selected dentists. The questionnaire was deemed to be easy to understand and easy to fill by participants in the pilot study.

All methods were performed in accordance with relevant guidelines and regulations.

The online application presented the retrieved data on an electronic spreadsheet. Data was analyzed with IBM SPSS Statistics for Windows, Version 21.0. Armonk, NY: IBM Corp.

Descriptive statistics were calculated for survey data. The level of statistical significance was set at *p* ≤ 0.05.

## Results

A total of 314 responses were recorded from 88 consultants/specialists, 28 private general practitioners, 43 public dental officers and 155 residents and house officers. Only 44 (14.0%) of the respondents work in a private setting while the rest of the respondents work in different types of public owned facilities. The gender ratio was 1.4:1 (M: F) and their ages ranged between 22 to 63 years with a mean age of 38.06 ± 8.9 years, the mean number of years since specialization (for consultants) was 6.21 ± 6.3 years with 1 to 33 years of work experience. Majority of the Specialists were oral and maxillofacial surgeons and most of the respondents practice in the south western region of Nigeria (Table 1).

**Table 1:** Demographics of participants

Gender	Male	184 (58.6%)
	Female	130 (41.4%)
Designation	Consultants/specialists	88 (28.0%)
	Private dental practitioners	28 (8.9%)
	Resident doctors	124 (39.5%)
	Dental officers	43 (13.7%)
	House officers	31 (9.9%)
Specialty	Oral and maxillofacial surgery	86 (38.9%)
	Family dentistry	33 (14.9%)
	Paediatric dentistry	26 (11.8%)
	Orthodontics	24 (10.9%)
	Restorative dentistry	18 (8.1%)
	Periodontology	12 (5.4%)
	Others	22 (9.9%)
Type of practice	Teaching hospital	205 (65.3%)
	Private hospital	44 (14.0%)
	General hospital	37 (11.8%)
	Federal medical centre	15 (4.8%)
	Military hospital	11 (3.5%)
	Mission hospital	2 (0.6%)
Location of practice	South west	175 (55.7%)
	North west	44 (14.0%)
	South south	41 (13.1%)
	North central	22 (7.0%)
	South east	19 (6.1%)
	North east	13 (4.1%)

**Fig. 1:**
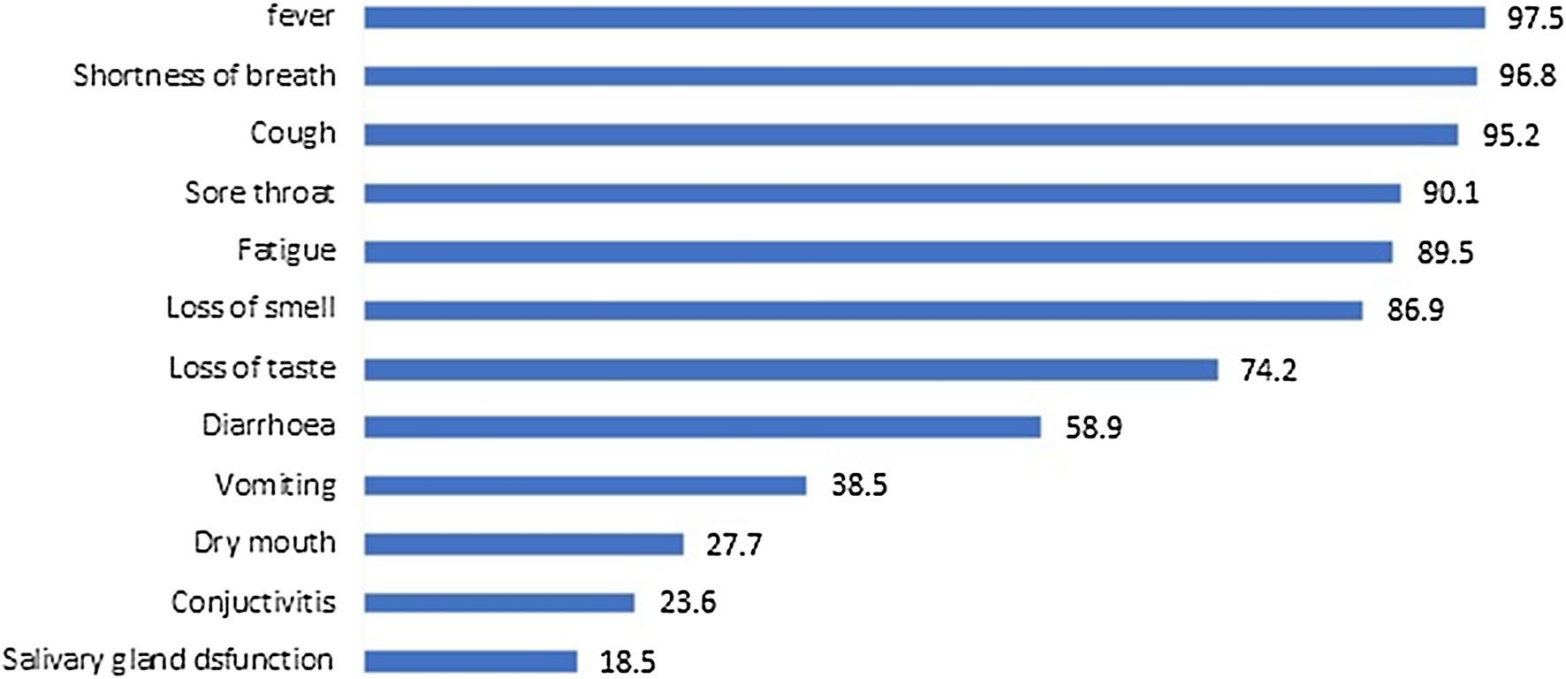
Symptoms associated with COVID-19 infection

Regarding participants’ knowledge of COVID-19 symptoms, fever was the most commonly specified generalized symptom (97.5%) (Fig. 1) while the use of face masks (100%), hand hygiene (99.7%), social distancing (97.7%) and surface cleaning (99.4%) were the most employed general preventive methods.

The main identified risk of transmission in the clinic was aerosol generating procedures (98.7%), followed by contaminated surfaces (98.1%), droplets (97.5%), Saliva (90.8%) and contaminated instruments (86.9%). Ultrasonic scaling was noted as the highest aerosol generating procedure (98.7%), followed by crown preparation (97.1%), third molar disimpaction (95.9%) and root canal therapy (85.7%). The respondents opined that routine extractions, impression making and orthodontic treatment are not aerosol generating procedures (AGPs).

Participants reported a significant increase in the deployment of teledentistry for appointment scheduling, consultation, conferencing and patient follow-up during the pandemic, as compared to before the COVID-19 outbreak (Table 2).

**Table 2:** Utilization of teledentistry before and during COVID-19 outbreak (n = 314)

Teledentistry	Before outbreak	During outbreak	*P*-value
	n (%)	n (%)	
Scheduling of appointment	123 (39.2)	165 (52.5)	< 0.001*
Consultation	26 (8.3)	88 (28)	< 0.001*
Conferencing	7 (2.2)	85 (27.1)	< 0.001*
Follow-up	101 (32.2)	242 (77.1)	< 0.001*

P-value obtained using McNemar test

*Significant at P < 0.05

One hundred and ninety-two participants (61.1%) stated that their clinics were closed at the inception of the outbreak. Of these, 38 (12.1%) shut down for over eight weeks while 15 (4.8%) remained closed as at the time of the survey. One hundred and eighteen respondents opened only to emergencies during the lockdown. The impact of clinics being closed was felt more in the south western (71.4%) and northern (63.3%) regions of the country, as compared to the south eastern and south southern regions (28.3%) (*p* < 0.001, Table 3). Moreover, the duration of closure was longest in the northern region, followed by the south eastern and south southern regions (*p* = 0.002). Regarding facility type, the proportion of clinics closed at the inception of the outbreak was about the same for private and public facilities (59.1% vs 61.5%; *p* = 0.76), however, the duration of closure was significantly longer in public facilities (*p* = 0.04), and a higher proportion of private clinic were open for business as at the time of this survey (83.3% vs 52%; *p* < 0.001) (Table 3).

**Table 3:** Association between impact variable and geographical regions, facility type

Geographical regions				Facility type	
Variables	North	SW	SE/SS	Private	Public
Closure at inception (n = 314)					
Yes	50 (63.3)	125 (71.4)	17 (28.3)	26 (59.1)	166 (61.5)
No	29 (36.7)	50 (28.6)	43 (71.7)	18 (40.9)	104 (38.5)
χ^2^ (*P*-value)	35.13 (< 0.001*)			0.09 (0.76)	
Duration of closure (n = 192)					
< 2 weeks	4 (8.0)	22 (17.6)	3 (17.6)	1 (3.8)	28 (16.9)
2 – 4 weeks	11 (22.0)	57 (45.6)	3 (17.6)	15 (57.7)	56 (33.7)
4 – 8 weeks	20 (40)	27 (21.6)	7 (41.2)	8 (30.8)	46 (27.7)
> 8 weeks	15 (30.0)	19 (15.2)	4 (23.5)	2 (7.7)	36 (21.7)
χ^2^ (*P*-value)	19.27 (0.002*^α^)			7.97 (0.04* ^α^)	
Current clinic status (n = 311)					
Full services	37 (47.4)	103 (59.5)	35 (58.3)	35 (83.3)	140 (52.0)
Emergency	41 (52.6)	70 (40.5)	25 (41.7)	7 (16.7)	129 (48.0)
χ^2^ (*P*-value)	3.33 (0.19)			14.50 (< 0.001*)	

SW: South West; SE: South East; SS: South South

*P *value obtained using Chi-square and ^α^Fisher’s exact test, *Significant at P < 0.05

**Fig. 2:**
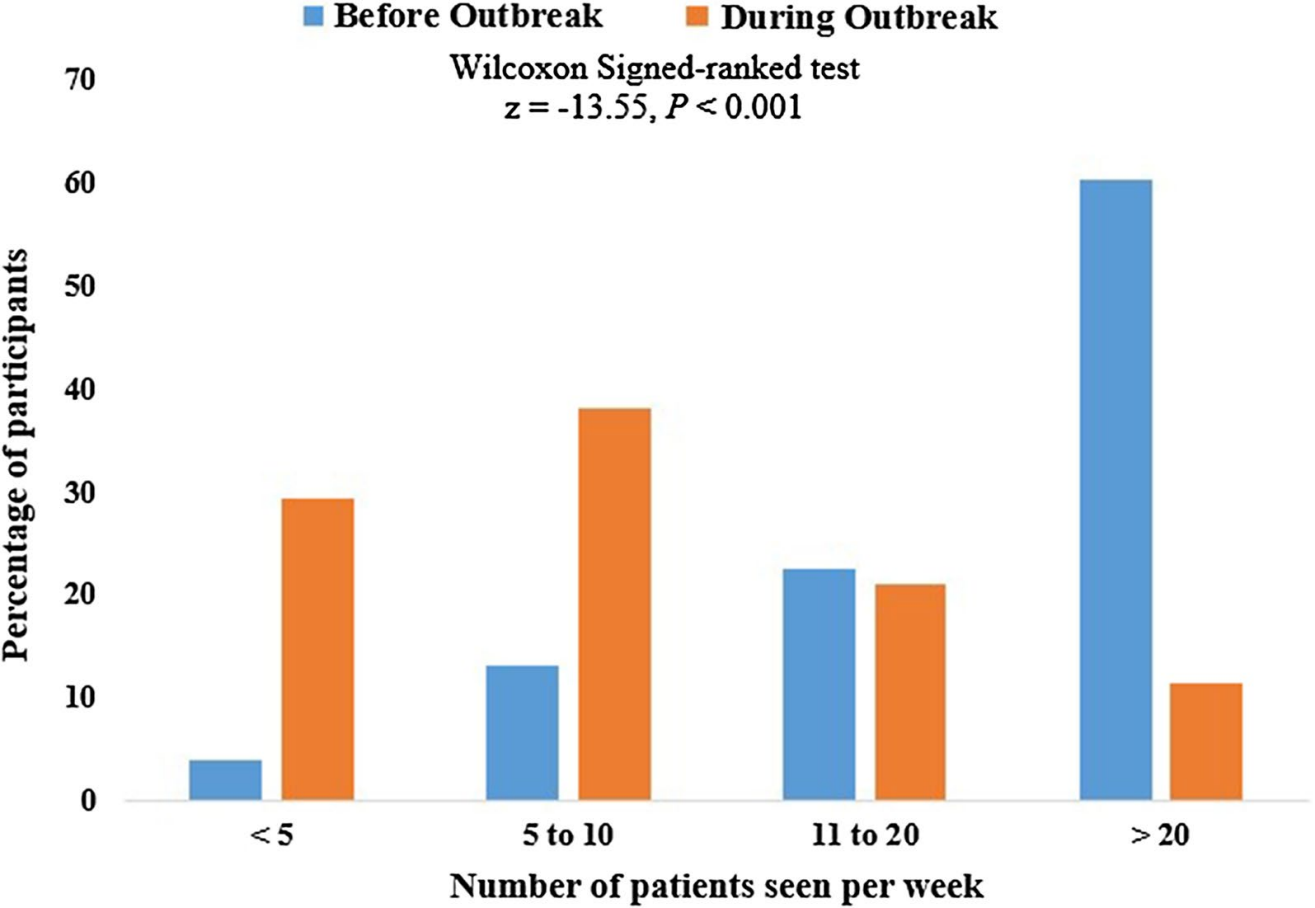
Number of patients attended to per week before and during COVID-19 Outbreak

Consequently, there was a reduction in the number of patients seen. Before the COVID-19 outbreak, 60% of the participants reported attending to more than 20 patients per week on the average, however at the time of this survey, only 11.5% were operating at such capacity (Fig. 2). A Wilcoxon Signed-ranked test determined that there was a statistically significant decrease in the median number of patients seen per week during the pandemic (5–10 patients) compared to before the pandemic (more than 20 patients): z = -13.55, *p* < 0.001 (Fig. 2). Pain remained the commonest reason for presentation before (169; 64.1%) and during (170; 64.4%) the pandemic (Table 4).

**Table 4:** Commonest reason for presentation (n = 264)

Reasons	Before COVID-19 outbreak	COVID-19 outbreak
	n (%)	n (%)
Pain	169 (64.1)	170 (64.4)
Routine check-up	46 (17.4)	52 (19.7)
Abscesses	19 (7.2)	17 (6.4)
Aesthetic concerns	18 (6.8)	15 (5.7)
Trauma	12 (4.5)	10 (3.8)
Total	264 (100.0)	264 (100.0)

As shown in Table 5, less than half of the participants (143; 45.5%) claimed to have received some formal training on infection prevention and control protocol for COVID-19 and the proportions did not differ significantly across the regions (*p* = 0.86) and nature of facility (*p* = 0.94). Furthermore, 209 (66.6%) dentists reported the presence of an infection prevention and control (IPC) focal person in their clinic. Although the proportions reported were relatively same across the regions (*p* = 0.13), they significantly differed across the facility type (*p* = 0.001) as more dentists in public facilities reported the presence of an IPC focal person.

**Table 5:** Association between preventive measures and geographical regions, facility type

Variables	Geographical regions			Facility type	
	North	SW	SE/SS	Private	Public
Formal training on IPC (n = 311)					
Yes	37 (48.1)	80 (46.0)	26 (43.3)	20 (45.5)	123 (46.1)
No	40 (51.9)	94 (54.0)	34 (56.7)	24 (54.5)	144 (53.9)
χ^2^ (*P*-value)	0.30 (0.86)			0.001 (0.94)	
IPC focal person (n = 311)					
Yes	48 (62.3)	121 (69.5)	40 (66.7)	24 (54.5)	185 (69.3)
No	12 (15.6)	21 (12.1)	14 (23.3)	15 (34.1)	32 (12.0)
Not sure	17 (22.1)	32 (18.4)	6 (10)	5 (11.4)	50 (18.7)
χ^2^ (*P*-value)	7.07 (0.13)			14.59 (0.001*)	
PPE donning & doffing (n = 311)					
Yes	33 (42.9)	68 (39.1)	24 (40)	17 (38.6)	108 (40.4)
No	44 (57.1)	106 (60.9)	36 (60)	27 (61.4)	159 (59.6)
χ^2^ (*P*-value)	0.32 (0.85)			0.05 (0.82)	
Sufficiency of PPE (n = 310)					
Insufficient	39 (50)	120 (69.8)	44 (73.3)	14 (31.8)	189 (71.1)
Somewhat	24 (30.8)	25 (14.5)	6 (10.0)	8 (18.2)	47 (17.7)
Sufficient	15 (19.2)	27 (15.7)	10 (16.7)	22 (50)	30 (11.3)
χ^2^ (*P*-value)	14.79 (0.01*)			42.63 (< 0.001*)	
Availability of PPE (n = 309)					
Not regular	40 (51.3)	92 (53.8)	43 (71.7)	9 (20.5)	166 (62.6)
Somewhat	28 (35.9)	57 (33.3)	8 (13.3)	17 (38.6)	76 (28.7)
Regular	10 (12.8)	22 (12.9)	9 (15)	18 (40.9)	23 (8.7)
χ^2^ (*P*-value)	10.27 (0.04*)			42.64 (< 0.001*)	
Protocols limiting no of personnel (n = 310)					
Yes	64 (84.2)	136 (78.2)	40 (66.7)	37 (84.1)	203 (76.3)
No	5 (6.6)	23 (13.2)	15 (25)	6 (13.6)	37 (13.9)
Not sure 7 (9.2)	15 (8.6)	5 (8.3)	1 (2.3)	26 (9.8)	
χ^2^ (*P*-value)	9.72 (0.05*)			2.62 (0.27 ^α^)	
Policies to reduce exposure (n = 311)					
Yes	47 (61)	107 (61.5)	30 (50)	32 (72.7)	152 (56.9)
No	13 (16.9)	32 (18.4)	18 (30)	9 (20.5)	54 (20.2)
Not sure	17 (22.1)	35 (20.1)	12 (20)	3 (6.8)	61 (22.8)
χ^2^ (*P*-value)	4.71 (0.32)			6.31 (0.04*)	
Screening bay/tent (n = 306)					
Yes	38 (50)	51 (29.8)	31 (52.5)	9 (20.5)	111 (42.4)
No	38 (50)	120 (70.2)	28 (47.5)	35 (79	151 (57.6)
χ^2^ (*P*-value)	14.43 (0.001*)			7.59 (0.01*)	

P-value was obtained using Chi-square or ^α^Fisher’s exact test

*Significant at P < 0.05

Of the participants, 240 (76.4%) responded that they have documented administrative protocols that aim at reducing the number of staff in the clinic per time (Table 5). Such administrative protocols were noted more in facilities situated in the northern region (84.2%) followed by the south western region (78.2%) and the south eastern and south southern regions (66.7%). The observed difference across private and public facilities was not statistically significant (*P* = 0.27), however, a higher proportion of respondents in private facilities opined that the protocol gives further protection to members of staff with identifiable vulnerability (72.7% vs 56.9%; *P* = 0.04). Table 5.

One hundred and twenty-five (39.8%) respondents have had demonstrations on safe donning and doffing of Personal Protective Equipment (PPE). The number of respondents who have had demonstrations on safe donning and doffing of Personal Protective Equipment (PPE) did not differ significantly across the regions (*p* = 0.85) and facilities (*p* = 0.82): only between 38 – 43% of the participants reported in the affirmative (Table 5). One hundred and twenty participants (38.2%) reported the availability of a dedicated screening bay or tent to attend patients on arrival. More than half of the respondents from the south eastern/south southern (52.5%) and northern region (50%) reported the availability of a dedicated screening bay or tent to attend patients on arrival. These were significantly higher compared to 29.8% from the south western region (*p* = 0.001). Moreover, availability of screening bay was noted more in public facilities compared to their private counterparts (42.4% vs 20.5%; *p* = 0.01). Although, 261 (83.1%) and 244 (77.7%) respectively screen for the presence of fever and ask about travel history as patients arrive, only 65 (20.7%) participants required their patients to complete a screening form. Two hundred and eighty-nine participants (92%) deferred treatment once a patient showed any symptoms suggestive of COVID-19. Hand hygiene station was marked as existing by 290 participants (90.3%) with many having multiple options for hand hygiene; alcohol rub (206, 65.6%) being the most common, followed by wash hand basins (204, 65.0%) and veronica buckets (134, 43.6%).

Furthermore, one hundred and eighteen respondents (37.6%) work in a clinic with 1–5 chairs and an overall median dental chair present in the clinics was 7. About two-fifth of the respondents (125; 39.8%) work in clinics with open floor models while about a third (98; 31.2%) and a quarter (79; 25.2%) of the respondents work in single room and hybrid model clinics respectively. Also, 57.7% (181) of the respondents reported that their waiting rooms have been rearranged to handle an average of 45% of their capacity. Concerning airflow solutions, 120 participants (38.2%) reported that their clinics have a definite airflow plan while only 18 (5.7%) and 27 (8.6%) reported presence of air filtration units and air exhaust in their clinics respectively. About 42% (132) of the respondents reported that they do not ask patients to perform any form of pre-procedural mouth rinse whereas, 29.3%, 24.2% and 4.6% utilise hydrogen peroxide, chlorhexidine and povidone iodine mouth rinses respectively.

**Fig. 3:**
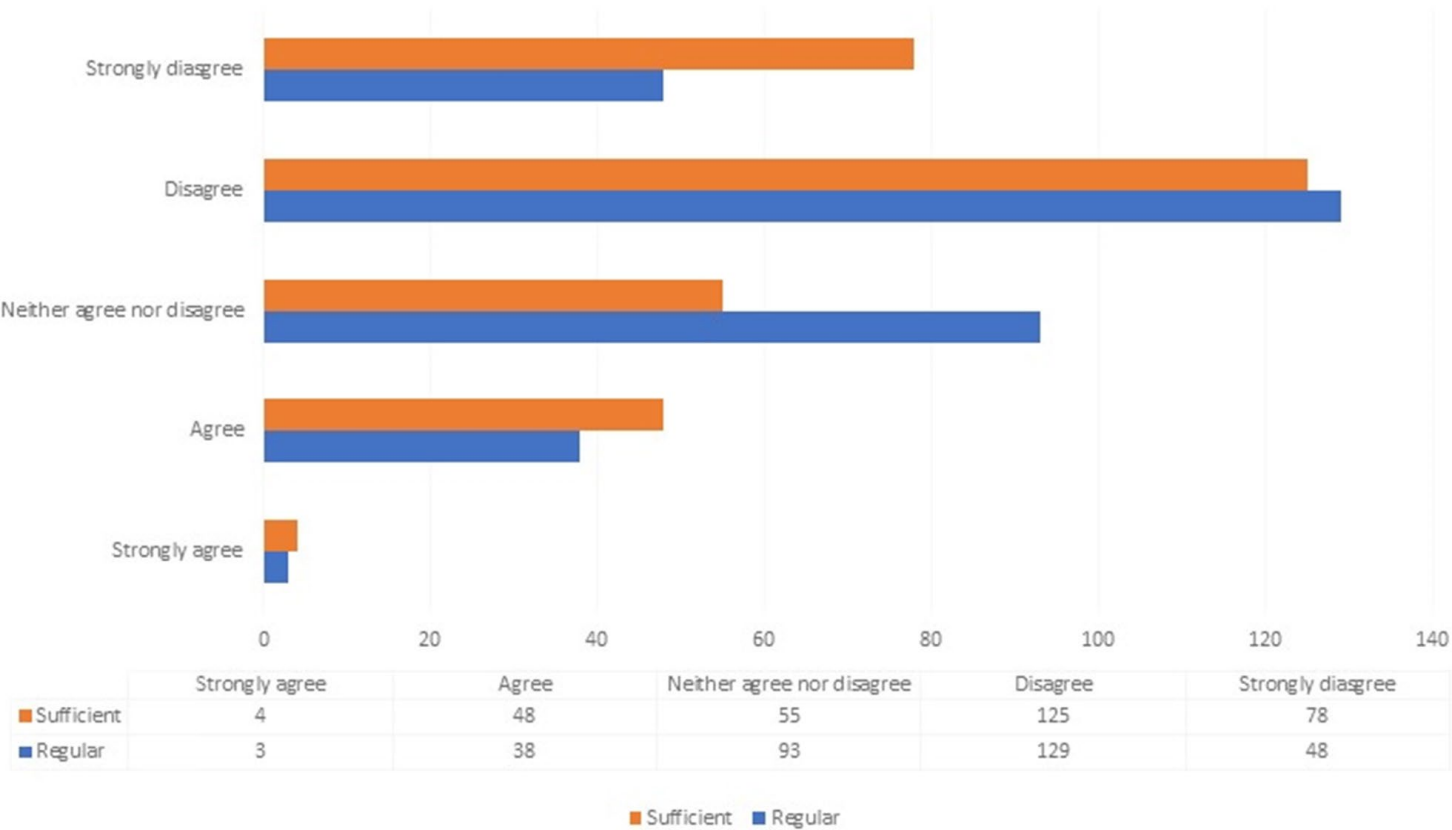
Sufficiency in the supply and availability of PPE

Concerning clinic outfit, there was a decrease in the use of ward coats in clinics with 39.6%, 43.4% and 17.0% wearing ward coats, scrubs, and uniforms respectively due to the outbreak, as against 71.3%, 18.3% and 10.4% prior to the outbreak. On the other hand, 282 respondents (89.8%) reported that surgical gloves and surgical masks were the most readily available PPE in the clinic, followed by face shields, conform gloves, and goggles (66.2%, 62.7% and 49.7%) respectively. Also, only 145 participants (46.1%) reported having fit tested the N95 mask with 112 (35.7%) and 31 (9.9%) participants reporting the availability of N95 and KN95 masks respectively. Concerning the sufficiency and availability of the PPE, most respondents reported that the supply was either insufficient or not regular(Fig. 3). Reported cases were significantly higher in the south eastern and south southern regions (73.3%; 71.7%), and south western region (69.8%; 53.8%), as compared to the northern region (50%; 51.3%). Furthermore, reported cases of PPE insufficiency and non-regular availability were significantly higher in public facilities (71.1%; 62.6%) compared to private facilities (31.8%; 20.5%).

## Discussion

This was a national survey intended to document the knowledge base, response and adaptation of Nigerian dentists and dental facilities to the novel COVID-19 pandemic since its advent in the country. For many reasons, it is not surprising that more than half (55.7%; 175) of the respondents practice in south-western Nigeria. Firstly, the index case in Nigeria was reported in Lagos, a south-western state which has become the epicentre of the disease. Coincidentally, the greater proportion of Nigerian dentists practice in the same geopolitical zone and most of the established accredited dental schools are also domiciled in the zone. It therefore stands to reason that a national survey of dentists in Nigeria would record the greatest participation from the south western region. In general, the survey yielded a wide and robust set of data which could be useful for understanding and planning a structured and co-ordinated dental healthcare management system in the pandemic and post pandemic era.

Respondents demonstrated good knowledge of the common symptoms of COVID-19 which include fever, dry cough, chest pain, dyspnoea, fatigue and myalgia. this is similar to the findings of Arora et al.[16] and Khader et al.[17] who reported good knowledge of COVID-19 among dentists in India and Jordan respectively.

In this study, less common symptoms such as headache, dizziness, nausea, vomiting, diarrhoea, abdominal pain and conjunctivitis were also less associated with COVID-19. In particular, fewer than 50% of participants knew about the association with vomiting, conjunctivitis and salivary gland disorders. However, it is crucial for dentists to know every and the least frequently documented symptom because of their high-risk position. The most intriguing fact about this novel disease is in the fact that it shares symptoms with many common illnesses, including nonspecific viral illnesses that are generally considered mild and self-limiting. A high index of suspicion is therefore required to avoid evitable exposure[18].

Person to person transmission of SARS-CoV-2 is the predominant mode of spread of the disease. In the dental environment, COVID-19 is transmitted mainly by: 1) direct transmission of the pathogen through inhalation of droplets generated when patients cough or sneeze; 2) direct transmission of pathogens via exposure of the ocular, oral or nasal mucous membranes to infectious droplets; and 3) indirect transmission of the pathogen via contaminated work surfaces[19]. Also, airborne transmission of SARS-CoV-2 can occur during aerosol generating dental procedures [14]. Thus, such procedures should only be done in well-ventilated rooms and with optimal personal protective equipment[20]. With aerosol-generating procedures being the main concern of the current challenge for dental services provision, interventions that avoid or limit aerosol generation should be the first choice. Such procedures may replace possibly unsafe “standard” therapies in an emergency situation with airborne pathogens such as SARS-CoV-2. Benzian and Niederman proposed the concept of Safer Aerosol-Free Emergent Dentistry (SAFERDentistry)[21]. SAFERDentistry relies on a prioritization of the most common patient needs and comprises of the selection of the most effective evidence-based, and value-based care that do not necessitate aerosol generating procedures. This is very important as the procedures do not require complex technology and are effective and achievable, even for resource-limited settings[22]. Furthermore, respiratory secretions, droplets or aerosols expelled by infected individuals can contaminate objects and surfaces. Reverse transcription polymerase chain reaction (RT-PCR) detectable viable SARS-CoV-2 and/or RNA can be found on surfaces ranging from hours to days following aerosol generating procedures. This depends on the ambience; including temperature, humidity, the type of surfaces and the concentrations of the emissions which are usually high in health care facilities where patients with COVID-19 are being treated[12]. Transmissions may therefore occur indirectly through touching contaminated surfaces and objects such as dental chair light handle/ switch, trays, chair adjustment buttons, workstation surfaces with subsequent transfer to the mucous membranes of the nose, mouth, or eyes. However, despite consistent evidence of surface contamination and fomite survival, there has not been a report of any specific case of SARS-CoV-2 infection arising from fomite transmission[11]. Nevertheless, it is recommended that all surfaces are cleaned properly with alcohol-based (70–90%), chlorine-based (5000 ppm), or hydrogen peroxide-based (> 0.5%) surface disinfectant after each patient's visit especially for high-touch surfaces and at least once daily terminal clean[23]. Contact time of a minimum of one minute or adherence to manufacturers’ instructions is recommended in the use of these disinfectants [11, 23].

It is noteworthy that most centres have reduced the number of patients accommodated in their waiting rooms but the maintenance of physical distance must still be emphasized and all patients in the reception area should use face masks[24].

However, only few clinics reported a definitive airflow plan. Definitive airflow is a major consideration in maintaining a safe workspace during the COVID-19 outbreak[25]. Ventilation engineering should be recognized as an important means of reducing airborne transmission as it controls how quickly room air is evacuated and changed over a period of time. Ventilation plays a key role in eliminating exhaled virus-laden air, thus dropping the overall concentration and any subsequent dose inhaled by the room occupants. This reduces the possible risk of transmission in any oral health care facility[26]. The use of split air conditioning systems or other types of recirculation devices should be circumvented, and facilities should consider fixing filtration systems or exhaust fans[27]. However, any modification of the ventilation system in an oral health care facility needs to be done with caution, taking into consideration the cost of the initial design, procurement, early and late maintenance and potential impact on the established airflow in other parts of the healthcare facility[28].

Every patient is potentially infectious as transmission of SARS-CoV-2 can occur in pre-symptomatic and asymptomatic stages[11]. In these situations, medical/travel history or body temperature measurements offer no guarantee of recognising an infected person. Furthermore, unavailability of reliable routine checks and valid point of care testing prior to dental care at this point, is another bane to prompt identification of infected individuals. The only safe haven is in the adoption of universal precautions. Every patient must be considered potentially infectious and should be treated with appropriate aseptic techniques and preventive barriers[11]. One of the findings in this study revealed that up to 17% of the studied population do not carry out temperature checks before admitting patients into the clinic. This may not be unconnected to the fact that the standard protocol of universal infection control is maintained in most of our facilities. However, vigilance in cases of obvious signs of respiratory symptoms must be instituted to prevent undue exposure to the virus.

It was generally agreed that hand hygiene is the first step in curtailing the spread of the virus; the WHO guidelines stipulate that meticulous hand-washing be performed before any aseptic procedure, before touching a patient, after touching a patient, after exposure to body fluids, and after touching a patient’s surroundings[11]. Also, patients should be requested to wash their hands before admittance into the clinic. Most of the participants in this survey prepared multiple hand hygiene options in their facilities. This shows the readiness of the oral health care personnel in forestalling the spread of the infection. However, with only about 65% of the respondents using wash hand basins and 10% with no hand hygiene measures at the clinic entrance, there is need for all dental clinics across the country to scale up their facilities and ensure adequate arrangement is made for hand hygiene measures.

The availability and sufficiency of PPE was a concern for majority of our respondents. It is important to emphasize that, regardless of the treatment planned, dental healthcare professionals must adhere to strict protocols related to clinic dressing and personal protective equipment. Hair caps, surgical gowns, surgical masks or N95, special foot wears, protective goggles, and protective visors are essential[12, 29]. The use of personal items should be minimised in the clinic as much as possible, instead, scrubs and clinic uniforms should be encouraged[19]. It is important that dentists, including all DHCP undertaking or assisting in procedures, are trained and understand how to properly donn, use, and doff appropriate PPE to prevent self-contamination[19]. A fit tested N95 or FFP2 respirator (or higher) is necessary when aerosol generating procedures (AGP) are performed, however, majority of our respondents have no fit tested N95 masks available for their work. This situation gives credence to the need for such participants to avoid AGPs.

Dental health care practitioners have a significant role in the worldwide fight against pandemics like COVID-19 because they are knowledgeable in cross-infection control procedures and barrier techniques. All dental care facilities, either privately owned or Government owned, should develop a simple-to-apply infection prevention and control protocol. The assignment of a focal person in charge of infection prevention and control is a laudable development as several of our participants alluded to this in their facilities. It is however worrisome that less than half of the respondents have received formal training on infection control concerning COVID–19 and less than 40% have had training on the rational use of PPE. It is imperative to ensure that dental health care personnel are formally trained in infection prevention and control, choice and appropriate use of Personal Protective Equipment (PPE) and in following a risk assessment and standard infection precautions. PPE include gloves, eye protection (face shield that covers the front and sides of the face or goggles), fluid resistant disposable gown, and a medical mask. This training needs to be taken as a major intervention strategy as less than half of our study participants have had such training.

This is the right time for dental schools to advance the learning outcomes of their courses to include added roles of dentistry that take into account natural disasters and pandemics preparedness.

During the COVID-19 pandemic, prevention of oral health problems and self-care remained of high importance. Information on keeping and maintaining good oral hygiene should be escalated to patients through remote consultations or/and social media channels. If and where possible, patients should be screened before their appointments either by virtual/remote technology or telephony[19].

Despite the increasing utilization of teledentistry in our study population, the use is still minimal compared to other countries[30, 31]. This may be related to unavailable or inaccessible uninterrupted internet supply in many parts of the country. Also, some patients may not be adequately technologically empowered to communicate with the clinician for tele-consultation, even when and where such facilities are available. Therefore, there is still a need to emphasize the importance of integration of health information management technology into patient management and to improve information technology infrastructure[32].

The participants in this study reported that majority of the clinics were partially or totally closed during the lockdown period in Nigeria. This is similar to the findings of the American Dental Association (ADA) Health Policy Institute (HPI) which showed that at the height of the pandemic in the United States, about 80% of dental practices offered only partial emergency services, and 17% of dental clinics did not see patients at all[33]. Furthermore, similar reports were received from other parts of the world as dental clinics were advised to close down or scale down activities as a preventive measure[19]. Many clinics were, however, already reopening at the time of this study.

According to WHO, routine oral health care such as oral health checks, scaling and polishing, aesthetic and preventive care should be deferred or carried out remotely until there has been appreciable decrease in COVID-19 transmission incidence from community transmission to cluster cases. However, the urgent oral health care services that are vital for preserving a functional oral complex, managing severe pain or maintaining quality of life that is essential should be provided.

Pre-procedural mouth rinses have been shown to reduce the oral viral load, since SARS-CoV-2 is sensitive to oxidation[14, 34]. Peng et al., proposed rinsing the mouth with 1% hydrogen peroxide or 0.2% povidone-iodine for 30 s before commencing a dental procedure[14]. However, over 40% of our respondents do not ensure any form of preprocedural mouth rinse. It is advised that this cheap and effective means of reducing oral pathogens be instituted in our clinics.

## Conclusion

The general knowledge of dentists in our study population appears to be adequate on the common features of COVID-19 but less adequate regarding the less common features. The COVID-19 pandemic has led to improvement in infection control protocol in most dental facilities in Nigeria.

Preventive measures to mitigate the spread of COVID-19 in the dental practice include proper utilization of teledentistry, clinical triage and screening, travel history, body temperature checks on arrival at the clinic, as well as preprocedural oral rinses with 1% hydrogen peroxide, and the use of appropriate PPEs should be encouraged at all times.

Also, pragmatic and technical recommendations for administrative and facility adaptation to ensure a safe space for the practice of dentistry should be incorporated into each facility, across the country.

### What is already known on this topic

- COVID-19 outbreak is a major test of the vulnerability of the global healthcare system.
- Dental healthcare provision is associated with increased risk of transmission of aerosol transmitted diseases.

### What this study adds

- Dentists in Nigeria have adequate knowledge of common symptoms of COVID-19 and understand the threat of disease transmission in the dental clinic setting.
- There is need for better facility disease prevention protocol development and facility adaptation to decrease risk of disease transmission in the dental clinic environment.

## Data Availability

The datasets used during this current study are available from the corresponding author on reasonable request.

## Abbreviations

H5N1: Highly pathogenic influenza virus
H1N1: Swine flu
SARS: Severe acute respiratory syndrome
MERS: Middle east respiratory syndrome
SARS-CoV-2: Severe acute respiratory syndrome coronavirus 2
WHO: World Health Organization
OHSA: Occupational safety and health administration
DHCP: Dental health care personnel
AGPs: Aerosol generating procedures
IPC: Infection prevention and control
PPE: Personal protective equipment
SAFERDentistry: Safer aerosol-free emergent dentistry
RT-PCR: Reverse transcription polymerase chain reaction
